# Data of H_2_O_2_ release from AQP8-knockdown rat hepatocyte mitochondria

**DOI:** 10.1016/j.dib.2019.103722

**Published:** 2019-03-06

**Authors:** Mauro Danielli, Julieta Marrone, Alejo M. Capiglioni, Raúl A. Marinelli

**Affiliations:** Instituto de Fisiología Experimental, Consejo Nacional de Investigaciones Científicas y Técnicas (CONICET), Facultad de Ciencias Bioquímicas y Farmacéuticas, Universidad Nacional de Rosario, Suipacha 570, 2000, Rosario, Santa Fe, Argentina

## Abstract

This article reports experimental data related to the research article entitled “Mitochondrial aquaporin-8 is involved in SREBP-controlled hepatocyte cholesterol biosynthesis” [Danielli et al., 2019]. We present data about hydrogen peroxide (H_2_O_2_) release from mitochondria isolated from rat hepatocytes with or without silencing of aquaporin-8 (AQP8) protein expression. The rate of mitochondrial H_2_O_2_ release (pmoles/min/mg mitochondrial protein) was found to be decreased by about 50% in AQP8-knockdown mitochondria.

**Table:** Specifications table

Subject area	Biology
More specific subject area	Mitochondrial biology
Type of data	Figure
How data was acquired	The assay utilizes horseradish peroxidase to catalyze the H_2_O_2_-dependent oxidation of non-fluorescent Amplex™ Red to fluorescent resorufin red and detects only the release of hydrogen peroxide, since the size of HRP prevents it from entering the mitochondria. Fluorescence was followed at a 565 nm wavelength every 3 min for 33 min at 37 °C in an automatic microplate reader (Beckman Coulter DTX 880 Multimode Detector) equipped with a thermally controlled compartment.
Data format	Analyzed data
Experimental factors	Freshly isolated rat hepatocytes were cultured and subjected to silencing of AQP8 protein expression and the mitochondria were isolated.
Experimental features	H_2_O_2_ release in isolated AQP8-knockdown mitochondria
Data source location	Rosario, Santa Fe, Argentina
Data accessibility	Data is available with this article
Related research article	M. Danielli, J. Marrone, A.M. Capiglioni, R.A. Marinelli. Mitochondrial aquaporin-8 is involved in SREBP-controlled hepatocyte cholesterol biosynthesis. Free Radic. Biol. Med. 2018 (in press) [1]

**Table:** 

**Value of the data** •The data highlight the role of the channel protein AQP8 as peroxiporin in hepatocyte mitochondria. •The data can be relevant in studies on hepatocyte redox-signaling. •The data can be useful in studies aimed to investigate mitochondrial oxidative stress.

## 1 Data

**Fig. 1:**
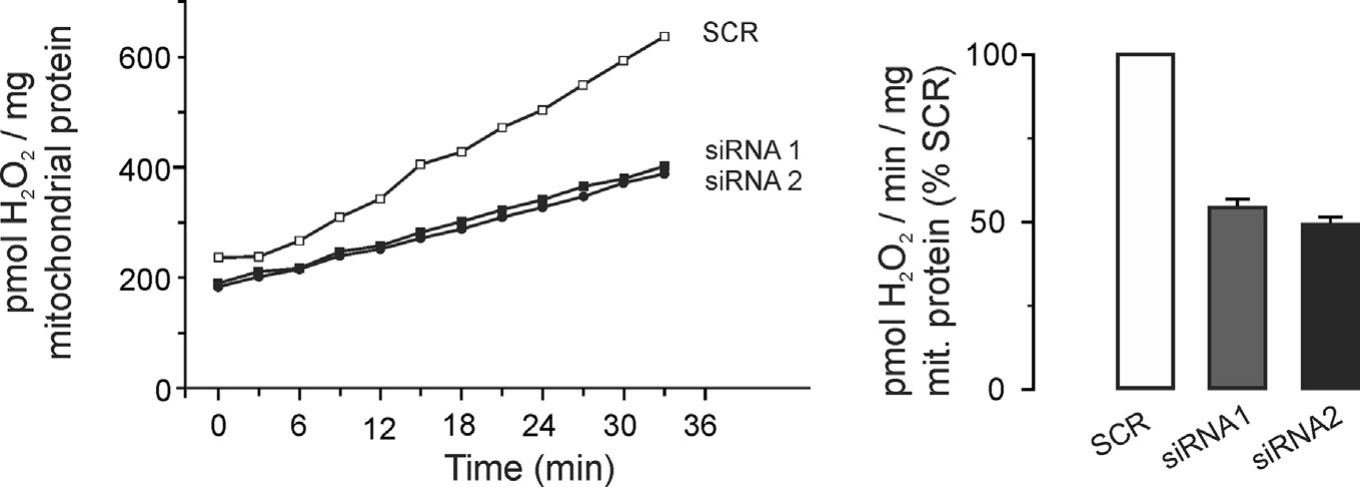
**H**_**2**_**O**_**2**_**release from AQP8-knockdown rat hepatocyte mitochondria**. Mitochondria were isolated from primary rat hepatocytes transfected for 24 or 48 h with siRNA specific for rat AQP8 or control scramble (SCR) (see Experimental design, materials and methods for details). The mtAQP8 protein expression was unaltered at 24 h but significantly decreased around 60% at 48 h [1]. *Left:* Time course of H_2_O_2_ release from mitochondria isolated from AQP8-knockdown hepatocytes (i.e., 48 h post-transfection). Data correspond to one of two independent experiments with similar results. *Right:* Rate of mitochondrial H_2_O_2_ release. Data are mean ± SEM of two independent experiments (siRNA1: 54% and siRNA2: 49%; expressed as percentage of scramble). At 24 h post-transfection with siRNAs or SCR, the rate of mitochondrial H_2_O_2_ release was unaltered (siRNA1: 105% and siRNA2: 99%; expressed as percentage of scramble; one of two independent experiments with similar results).

Here we show data of H_2_O_2_ release in mitochondria isolated from primary rat hepatocytes with or without knockdown of mitochondrial AQP8 (mtAQP8) protein expression (Fig. 1, left). The rate of mitochondrial H_2_O_2_ release (pmoles/min/mg mitochondrial protein) showed a reduction of about 50% in mitochondria from mtAQP8-knockdown hepatocytes (Fig. 1, right).

## 2 Experimental design, materials and methods

### 2.1 Materials and reagents

Dulbecco's Modified Eagle Medium, Pen-Strep antibiotic mixture, l-glutamine, and Lipofectamine 2000 Reagent were all from Invitrogen Corp., CA, USA Foetal Calf Serum were purchased from Internegocios S.A. laboratories, Bs As, Argentina. Silencer siRNA Construction kit was from Ambion, TX, USA, whilst collagenase type IV was from Sigma AldrichSigma, MO, USA as well as the protease inhibitor Phenyl-methylsulfonyl fluoride. Leupeptin was from Chemicon Millipore (Darmstadt, Germany). Amplex™ Red hydrogen peroxide/peroxidase assay kit was from Promega.

### 2.2 Isolation and culture of rat hepatocytes

Hepatocytes were isolated from normal livers of male Wistar rats by collagenase perfusion and mechanical disruption [2]. Cell viability (assessed by Trypan blue exclusion) was >85%. Hepatocytes were plated onto collagen-coated glass plates at 1.9 × 10^4^ cells/cm^2^. Primary rat hepatocytes were cultured in Dulbecco's Modified Eagle Medium (4.5 g/l), supplemented with 2 mM l-glutamine, 10% heat-inactivated foetal calf serum and 100 I.U. penicillin/100 μg streptomycin at 37 °C in a 5% CO2 atmosphere. Media was changed every other day.

### 2.3 Synthesis of short interfering RNA (siRNA) and AQP8 knockdown

As we previously reported [2], [3], the 21-nucleotide RNA duplexes were synthesized using the Silencer siRNA kit following the manufacturer's directions, with oligonucleotides synthesized by Invitrogen as templates. The siRNA1 and siRNA2 were targeted to two different regions of the rat AQP8 molecule. Corresponding control siRNA (SCR) was designed by randomly scrambling the nucleotides of siRNA1 [1], [2]. After 18 h of culture, hepatic cells were transfected with siRNAs by using Lipofectamine 2000 transfection reagent following the manufacturer instructions. After 24 and 48 h of transfection, cells were sonicated in 0.3 M sucrose containing 0.1 mM phenylmethanesulfonyl fluoride and 0.1 mM leupeptin and a 6000×*g* postnuclear mitochondrial fractions was prepared [2], [3]. Hepatocyte viability assessed by lactate dehydrogenase leakage was unaffected after at 24 or 48 h of transfection with siRNAs [1].

### 2.4 Mitochondrial H_2_O_2_ release in isolated mitochondria

H_2_O_2_ release from isolated mitochondria was measured by using the Amplex™ Red-horseradish peroxidase assay kit as previously described [3].

## References

[1] Danielli M., Marrone J., Capiglioni A.M., Marinelli R.A. (2019). Mitochondrial aquaporin-8 is involved in SREBP-controlled hepatocyte cholesterol biosynthesis. Free Radic. Biol. Med..

[2] Soria L.R., Marrone J., Calamita G., Marinelli R.A. (2013). Ammonia detoxification via ureagenesis in rat hepatocytes involves mitochondrial aquaporin-8 channels. Hepatology.

[3] Marchissio M.J., Francés D.E.A., Carnovale C.E., Marinelli R.A. (2012). Mitochondrial aquaporin-8 knockdown in human hepatoma HepG2 cells causes ROS-induced mitochondrial depolarization and loss of viability. Toxicol. Appl. Pharmacol..

